# Potential clinical and economic benefits of remote deep brain stimulation programming

**DOI:** 10.1038/s41598-022-22206-z

**Published:** 2022-10-19

**Authors:** Dávid Pintér, Evelyn Járdaházi, József Janszky, Norbert Kovács

**Affiliations:** 1 Department of Neurology, Medical School, University of Pécs, 7623, Pécs, Rét Utca 2, Pécs, Hungary; 2 ELKH-PTE Clinical Neuroscience MR Research Group, Pécs, Hungary

**Keywords:** Movement disorders, Parkinson's disease, Dystonia

## Abstract

Deep brain stimulation (DBS) teleprogramming may help reducing travel-related and other financial burdens for patients and maintaining DBS care in special situations. To determine travel-related burdens of DBS patients and explore effects of COVID-19 on DBS care. Travel- and visit-related data of 319 patients were retrospectively analyzed for the first year, five years, and ten years after initiating DBS. Frequencies of in-person and telemedicine visits over the 18-month periods just before and after the outbreak of COVID-19 in Hungary were also compared. Average travel distance during an in-person visit was 415.2 ± 261.5 km, while average travel time was 342.1 ± 199.4 min. Travel costs for the first year, five years, and ten years were 151.8 ± 108.7, 461.4 ± 374.6, and 922.7 ± 749.1 Euros, respectively. Travel distance, age, and type and severity of disease could help identify patients who would particularly benefit from teleprogramming. We detected a significant decrease in the number of visits during COVID-19 pandemic (from 3.7 ± 2.1 to 2.4 ± 2.7; *p* < 0.001) which mainly resulted from the decreased frequency of in-person visits (3.6 ± 2.0 vs. 1.7 ± 1.8; *p* < 0.001). Our results support the introduction of DBS teleprogramming in Hungary which could save money and time for patients while maintaining a secure delivery of DBS.

## Introduction

Deep brain stimulation (DBS) has revolutionized the treatment of movement disorders (MD) resistant to pharmacotherapy^1–3^. Recently, DBS systems have undergone a remarkable technical evolution. Among several innovations, development of remotely programmable systems has been a considerable step towards resolving several issues of postoperative care^4–10^. First, neurostimulation therapy is usually available only in highly specialized, mostly tertiary MD centers. Therefore, many patients who are treated with DBS have to travel long distances to receive medical care which can impose high expenses and inconvenience on patients and take much of their time. Furthermore, long-distance travels may be challenging for patients with mobility issues and require the help of caregivers. However, especially after the initiation of the stimulation and as the disease progresses, reprogramming of the DBS device may be necessary from time to time. In many cases, the caregivers have to take a day off from their workplaces to be able to accompany the patients for these visits. Second, because of the long distance between the home of the patient and the center, the resolution of stimulation- and device-related problems may take a longer time than optimal. It is sometimes also difficult to keep to appointment times and patients, therefore, need to spend more time than planned at the clinic. Finally, some patients who would be eligible candidates for DBS treatment may refuse to undergo the surgery because of the long-distance, time-consuming, and costly travels after the initiation of stimulation.

The COVID-19 pandemic has accelerated the digitalization of the healthcare. Telemedicine has been introduced in many countries, also including Hungary. In cases of the approximately 1000 Hungarian patients with movement MDs treated with DBS, only teleprogramming has been missing to fully complete all major aspects of neurological care. During COVID-19, remote programming has already been shown an efficient and timely procedure for maintaining the delivery of DBS therapy^4–7^. Among the producers of DBS devices, Abbott has been the first introducing a new technology (NeuroSphere™) allowing patients to communicate with their neurologists and receive DBS adjustments from home or another location with WiFi or cellular access. This innovation was approved by the Food and Drug Administration and got CE mark, after which it has been started being used in the United States^9^ and in several European countries such as the United Kingdom, Ireland, and Germany.

According to the first experiences with remote DBS programming and some theoretical considerations, teleprogramming may have several clinical and economic benefits. Among others, teleprogramming could save time and money for both patients with DBS and their caregivers. To in-depthly examine this potential advantage of teleprogramming and explore factors that could help identify patients who would benefit from teleprogramming to a greater extent, we conducted a single-center, registry-based study analyzing data of all patients who are being treated with DBS in our unit. Additionally, because teleprogramming has been found suitable for maintaining DBS care during COVID-19 in some pilot studies, we also tried to explore the effects of the pandemic on DBS care focusing on patients with MDs in Hungary.

## Materials and methods

All study-related procedures were approved by the National Ethical Board of Hungary and the National Institute of Pharmacy and Nutrition (031037/2015/OTIG and OGYÉI/6916/2021). The study was performed in accordance with the Declaration of Helsinki.

### Study population

All included patients fulfilled the following inclusion criteria: the subject (1) must have signed an informed consent form approved by the respective Ethical Committees prior to the inclusion in our MD registry; (2) received DBS for a medication-refractory disorder approved as an indication for DBS; (3) had his/her DBS system activated in our center; (4) had at least a one-year follow-up in our unit after the activation, and (5) had at least one in-person visit in our unit during the first year after the activation.

### Sources of data

The present study was based on the prospective MD registry of the Movement Disorder Unit of the Department of Neurology, University of Pécs which is a tertiary MD center in Hungary. The registry was approved by the Hungarian Medical Research Council and fulfills all security and data protection requirements. It was used to identify patients eligible for the analyses, and it also served as the source of disease-specific data (e.g., severity of disease) recorded during the DBS activation in-patient visit.

Additional data that were necessary to the analyses (e.g., address of the patient, number of visits in different years) were collected using healthcare information systems that are easily accessible by clinicians and include out- and in-patient visits, in-person and telemedicine visits, diagnostic procedures (e.g., lab tests, radiological examinations), drug prescriptions and refills taking place in both state-funded and private institutions.

Travel-related outcomes were modeled estimates. To determine the travel distances and times between the homes of patients and our MD unit, the route planer called ViaMichelin (https://www.viamichelin.com) was used. In all cases, the fastest routes were considered. In searching options, hatchback was selected for type of car and E5 unleaded petrol for type of fuel. Travel costs were calculated according to the instructions of the National Tax and Customs Administration of Hungary (https://en.nav.gov.hu). At the time of the analyses, one Hungarian Forint was 0.0028 Euro, while petrol cost for one kilometer travel was 35.7 Hungarian Forints (0.1 Euro). In addition to fuel costs, road tolls were also included in total travel costs.

During the analyses, only such in-person visits were taken into account where programming changes were performed which could have not been made without an in-person meeting between the patient and the doctor. These included changing the lower and/or upper limits of patient amplitude control at least, however, also more complex adjustments (e.g., changing the contact used for stimulation, the frequency and pulse width of the stimulation or creating a completely new program) in some cases.

### Primary endpoints

The main outcomes of the analysis included.

1. The average travel distance from home to our center and from our unit back to home per visit;
2. The average travel time per visit;
3. The average number of in-person visits during the first year, five years and ten years after the activation;
4. The average total travel time during the first year, five years and ten years after the activation; and
5. The average number of days caregivers have to take off from their workplaces to be able to accompany the patients for visits during the first year, five years and ten years after DBS activation.

Because the COVID-19 pandemic and consequent restrictions might have had an impact on the frequency of on-site visits, data from the period within one year before and the years of the pandemic were not taken into account during the analyses for primary endpoints 3, 4 and 5. In-person visit numbers for 5 and 10 years were determined with projection using the numbers of all in-person visits and the follow-up period on patient level. Primary outcome data were determined for both the whole study population and disorder subgroups [Parkinson’s disease (PD), dystonia, and essential tremor (ET)]. Subgroup data were afterwards compared.

### Secondary endpoints

In the present study,

1. Average travel costs per visit, and
2. Average cumulative travel costs during the first year, five years and ten years after the activation were also determined.

Data from the period within one year before and the years of the pandemic were not taken into account during the analyses for secondary endpoint 2 because of suspected effects of COVID-19 pandemic on the number of in-person visits. Secondary outcome data were determined for both the whole study population and disorder subgroups (PD, dystonia, and ET). Subgroup data were afterwards compared.

### Tertiary endpoints

We also tried to

1. Determine factors that may help the selection of patients who could benefit from remote programming to a greater extent by comparing data on in-person visits between patients.
  - Representing different disorder subgroups,
  - Living ≤ 50 km and further than 50 km from our unit,
  - Aged ≤ 65 years and over 65 years,
  - Having a level of education ≤ 12 years and more than 12 years, and.
  - Representing different levels of disease severity according to the Motor Part of the Movement Disorder Society-sponsored Unified Parkinson’s Disease Rating Scale (MDS-UPDRS)^11^, the Hoehn and Yahr Scale (HYS)^12^, the Burke-Fahn-Marsden Dystonia Disability Scale (BFMDRS)^13^, and the summary index of the Quality of Life in Essential Tremor Questionnaire^14^ assessed during the DBS activation in-patient visit; and
2. Assess the effects of COVID-19 pandemic on DBS care, by comparing.
  - The number of visits over 18 months just before (from October 1, 2018 to March 10, 2020) and after (from March 11, 2020 to September 8, 2021) the declaration of emergency due to COVID-19 pandemic in Hungary (March 11, 2020) independently of the type of visit (in-person or telemedicine visit), and
  - The numbers of in-person and telemedicine visits considering the same periods before and after the outbreak of the COVID-19 pandemic in Hungary.

For tertiary endpoint 2, only data of patients who had their implanted DBS systems activated at least 18 months before the outbreak of the pandemic were considered.

### Statistical analysis

Depending on sample size, to test normality, the Kolmogorov–Smirnov test or the Shapiro–Wilk test was used. Because analyzed data did not follow the normal distribution, for comparing disorder subgroup and travel distance-, age-, education- and disease severity-related data, Mann–Whitney U tests, while for comparing visit numbers before and after the outbreak of COVID-19 pandemic, Wilcoxon tests were used. To analyze correlations between travel distance and numbers of in-person visits and correlations between disease severity and numbers of in-person visits, Spearman’s rank correlations were used. The level of statistical significance was set at 0.05. All statistical analyses were performed using jamovi version 2.2.5.

## Results

### Demographics

Our MD registry included 319 patients who fulfilled all eligibility criteria. They received DBS mostly for PD (67.1%), dystonia (22.8%), and ET (5.3%, Table 1). Of them, only 54 patients (16.9%) were living in Baranya county in which Pécs lies, while these numbers were 36 (16.8%), 10 (13.7%), and 7 (41.2%) subjects for PD, dystonia, and ET patients, respectively.

**Table 1:** Travel- and visit-related data.

	All	PD	Dystonia	ET	ET-PD	Other types of tremor^a^	Other^b^
Number of patients	319 (100.0)	214 (67.1)	73 (22.8)	17 (5.3)	2 (0.6)	4 (1.3)	9 (2.8)
Follow-up period (years)	5.8 ± 3.4 [1.0, 18.2]	5.4 ± 3.1 [1.0, 15.6]	6.9 ± 3.5 [1.3, 14.5]	6.1 ± 4.9 [1.2, 18.2]			
At least 1-year follow-up	319 (100.0)	214 (100.0)	73 (100.0)	17 (100)			
At least 2-year follow-up	279 (87.5)	185 (86.4)	67 (91.8)	13 (76.5)			
At least 5-year follow-up	169 (53.0)	103 (48.1)	48 (65.8)	9 (52.9)			
At least 10-year follow-up	36 (11.3)	16 (7.5)	14 (19.2)	4 (23.5)			
Distance between home and the clinic (km)^c^	207.6 ± 130.8 [10, 544]	197.7 ± 122.5 [10, 521]	244.7 ± 141.0 [10, 520]	175.8 ± 164.1 [10, 501]			
1–100 km	87 (27.3)	62 (29.0)	13 (17.8)	7 (41.2)			
101–200 km	47 (14.7)	33 (15.4)	11 (15.1)	2 (11.8)			
201–300 km	126 (39.5)	84 (39.3)	29 (39.7)	5 (29.4)			
301–400 km	29 (9.1)	21 (9.8)	7 (9.6)	1 (5.9)			
401–500 km	23 (7.2)	10 (4.7)	12 (16.4)	1 (5.9)			
501–600 km	7 (2.2)	4 (1.9)	1 (1.4)	1 (5.9)			
Total distance travelled from home to the clinic and from the clinic to home (km)	415.2 ± 261.5 [20, 1088]	395.4 ± 244.9 [20, 1042]	489.3 ± 282.0 [20, 1040]	351.7 ± 328.3 [20, 1002]			
Travel time between home and the clinic (min)^c^	169.9 ± 96.5 [15, 521]	163.7 ± 92.4 [15, 432]	197.3 ± 106.8 [15, 521]	153.9 ± 133.8 [15, 417]			
1–60 min	66 (20.7)	47 (22.0)	10 (13.7)	7 (41.2)			
61–120 min	37 (11.6)	24 (11.2)	7 (9.6)	2 (11.8)			
121–180 min	44 (13.8)	31 (14.5)	11 (15.1)	1 (5.9)			
181–240 min	105 (32.9)	76 (35.5)	21 (28.8)	3 (17.6)			
241–300 min	36 (11.3)	22 (10.3)	11 (15.1)	1 (5.9)			
301–360 min	23 (7.2)	12 (5.6)	10 (13.7)	1 (5.9)			
361–420 min	5 (1.6)	1 (0.5)	2 (2.7)	2 (11.8)			
421–480 min	2 (0.6)	1 (0.5)	1 (1.4)	0 (0.0)			
481–540 min	1 (0.3)	0 (0.0)	0 (0.0)	0 (0.0)			
Total travel time from home to the clinic and from the clinic to home (min)	342.1 ± 199.4 [30, 1042]	327.4 ± 184.8 [30, 864]	394.5 ± 213.5 [30, 1042]	307.8 ± 267.5 [30, 834]			
Travel cost between home and the clinic (Euros)^c^	29.7 ± 15.9 [1.0, 65.1]	28.7 ± 15.1 [1.0, 62,8]	33.7 ± 16.7 [1.0, 62.7]	23.9 ± 20.9 [1.0, 60.8]			
1–10 Euros	54 (16.9)	36 (16.8)	10 (13.7)	7 (41.2)			
10.1–20 Euros	31 (9.7)	25 (11.7)	3 (4.1)	0 (0.0)			
20.1–30 Euros	42 (13.2)	29 (13.6)	9 (12.3)	2 (11.8)			
30.1–40 Euros	127 (39.8)	84 (39.3)	30 (41.1)	5 (29.4)			
40.1–50 Euros	35 (11.0)	26 (12.1)	8 (11.0)	1 (5.9)			
50.1–60 Euros	19 (6.0)	10 (4.7)	9 (12.3)	0 (3.7)			
60.1–70 Euros	11 (3.4)	4 (1.9)	4 (5.5)	2 (11.8)			
Total travel cost from home to the clinic and from the clinic to home (Euros)	50.4 ± 28.8 [2.0, 119.5]	48.4 ± 27.2 [2.0, 114.9]	58.2 ± 30.6 [2.0, 114.7]	41.5 ± 37.1 [2.0, 110.9]			
Total time spent on an in-person visit without detailed status assessment (min)^d^	452.1 ± 199.4 [140, 1152]	437.4 ± 184.8 [140, 974]	504.6 ± 213.5 [140, 1152]	417.8 ± 267.5 [140, 944]			
Total time spent on an in-person visit with detailed status assessment (min)^d^	542.1 ± 199.4 [230, 1242]	527.4 ± 184.8 [230, 1064]	594.6 ± 213.5 [230, 1242]	507.8 ± 267.5 [230, 1034]			

Data are n (%) or mean ± standard deviation [minimum, maximum].

ET = essential tremor; ET-PD = coincidence of essential tremor and Parkinson’s disease; PD = Parkinson’s disease.

^a^Other types of tremor include dystonic tremor (n = 1, 0.3%), Holmes tremor (n = 1, 0.3%), tremor related to spinocerebellar ataxia (n = 1, 0.3%), and tremor related to multiple sclerosis (n = 1, 0.3%).

^b^Other includes epilepsy (n = 6, 1,9%), Tourette’s syndrome (n = 1, 0.3%), autism (n = 1, 0.3%), and poststroke hyperkinesia (n = 1, 0.3%).

^c^Data refer for a one-way travel from the home of patients to our unit.

^d^This includes time of travelling from home to the clinic, time spent at the clinic and time of travelling home from the clinic.

**Figure 1:**
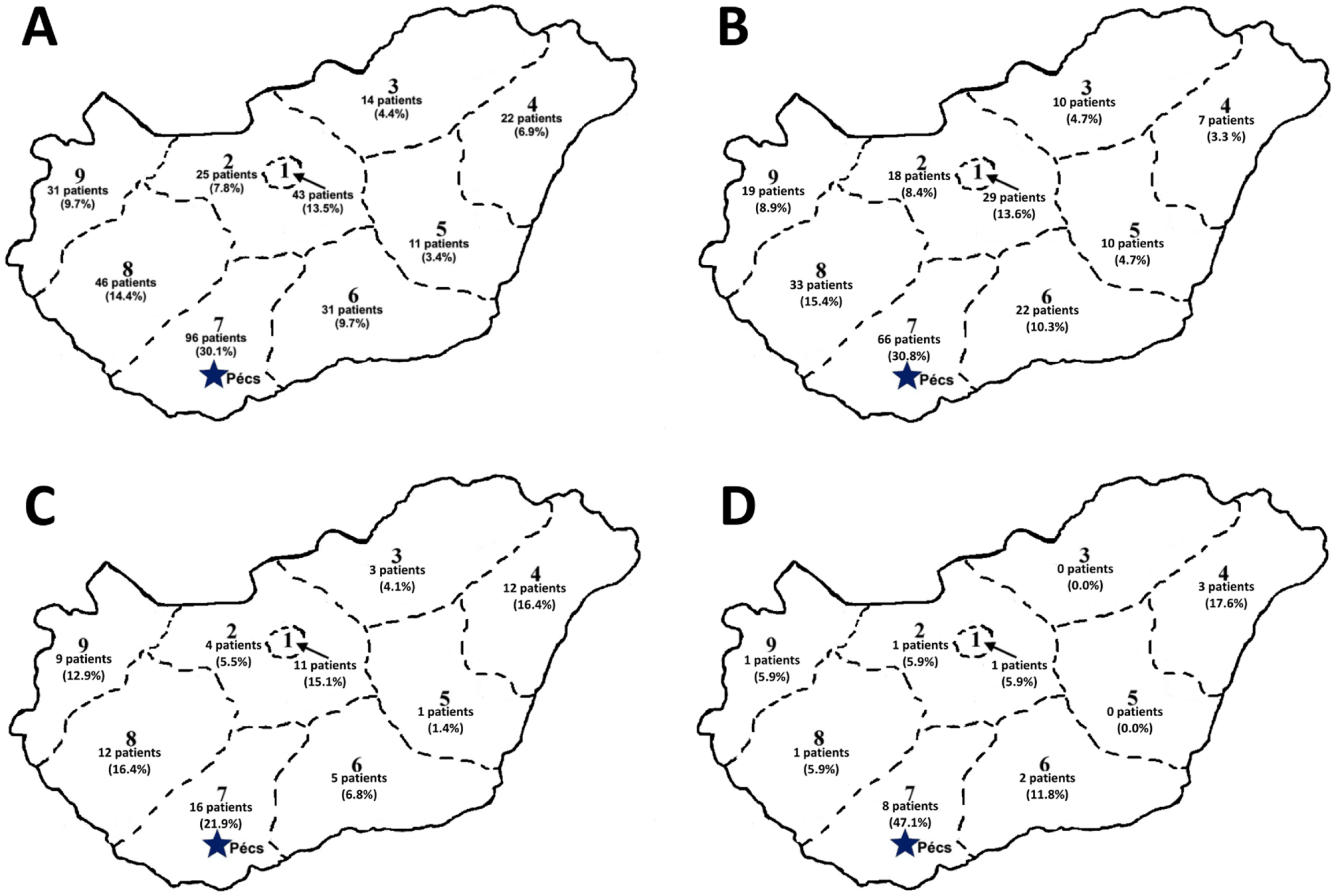
Numbers of patients from different postal code areas of Hungary regarding all patients (**A**) and subjects with Parkinson’s disease (**B**), dystonia (**C**), and essential tremor (**D**).

Follow-up visits took place between March 3, 2003 and September 8, 2021. The mean follow-up period was 5.8 ± 3.4 years (a minimum of 1.0 year and a maximum of 18.2 years). Of all patients, 279 (87.5%), 169 (53.0%), and 36 (11.35) subjects had a follow-up period of at least 2 years, 5 years, and 10 years, respectively (Table 1). Our patient pool treated with DBS was covering the whole country (Fig. 1).

### Primary endpoints

Based on data of the whole study population, the average distance patients with DBS and their caregivers had to travel from home to our center and from our unit back to home per visit was 415.2 ± 261.5 km, while the average time of this travel was 342.1 ± 199.4 min (Table 1). For 31 patients (9.7%), total time needed for an in-person visit without detailed status assessment was more than 12 h, while the number of patients who had to spent more than 12 h for in-person visits with detailed status assessment was 55 (17.2%).

Analyzing data of 298 patients, the average number of in-person visits during the first year after DBS activation was 3.3 ± 1.7, while these numbers projected for five and ten years were 10.5 ± 7.0 and 21.0 ± 14.0, respectively. The average times our patients with DBS and their caregivers have to spend on travel from home to our center and from our unit back to home to have their in-person visits during the first year, five years, and ten years after the activation are 17.2 ± 12.1 h, 52.2 ± 42.2 h, and 104.4 ± 84.3 h, respectively. The average number of days caregivers have to take off from their workplaces to be able to accompany the patients for these visits are 3 days, 9 days, and 17 days during the first year, five years, and ten years after the activation, respectively. However, in some cases, these numbers were found 9 days, 32 days, and 63 days for the first year, five years, and ten years, respectively. Disorder subgroup outcomes for primary endpoints can also be found in Tables 1 and 2.

**Table 2:** Visit-related data for the first year, five years and ten years after the activation.

	All (n = 298)	Parkinson’s disease (n = 195)	Dystonia (n = 72)	Essential tremor (n = 17)
**Data for the first year**				
Number of in-person visits in the first year	3.3 ± 1.7 [1, 10)	3.2 ± 1.7 [1, 10)	3.7 ± 1.9 [1,10]	2.4 ± 1.5 [1,6]
1 in-person visit in the first year	34 (11.4)	21 (10.8)	6 (8.3)	6 (35.3)
2 in-person visits in the first year	67 (22.5)	48 (24.6)	11 (15.3)	4 (23.5)
3 in-person visits in the first year	87 (29.2)	52 (26.7)	27 (37.5)	3 (17.6)
4 in-person visits in the first year	49 (16.4)	34 (17.4)	12 (16.7)	3 (17.6)
5 in-person visits in the first year	22 (7.4)	14 (7.2)	7 (9.7)	0 (0.0)
6 in person visits in the first year	23 (7.7)	18 (9.2)	3 (4.2)	1 (5.9)
7 in-person visits in the first year	7 (2.3)	4 (2.1)	2 (2.8)	0 (0.0)
8 in-person visits in the first year	4 (1.3)	2 (1.0)	2 (2.8)	0 (0.0)
9 in-person visits in the first year	2 (0.7)	1 (0.5)	1 (1.4)	0 (0.0)
10 in-person visits in the first year	2 (0.7)	1 (0.5)	1 (1.4)	0 (0.0)
Time spent on travel from home to the clinic and from the clinic to home during in-person visits in the first year (hours)	17.2 ± 12.1 [0.5, 83.4]	16.0 ± 10.7 [1.5, 63.0]	21.4 ± 14.5 [0.9, 83.4]	11.3 ± 10.3 [0.5, 28.7]
Total time spent on in-person visits in the first year (hours)^a^	26.1 ± 17.3 [3.8, 102.9]	24.9 ± 15.3 [4.9, 77.0]	30.9 ± 20.5 [4.2, 102.9]	18.2 ± 15.6 [3.8, 39.0]
Total working time lost in the first year (hours)	21.4 ± 10.1 [3.8, 72.0]	20.9 ± 9.4 [4.9, 55.8]	24.1 ± 11.1 [4.2, 72.0]	14.1 ± 8.1 [3.8, 32.0]
Total travel cost in the first year (Euros)	151.8 ± 108.7 [2.0, 726.3]	141.8 ± 97.2 [6.0, 591.0]	190.0 ± 130.0 [4.6, 726.3]	88.5 ± 85.2 [2.0, 226.8]
**Data for five years**				
Number of in-person visits over five years	10.5 ± 7.0 [1, 57]	10.6 ± 6.7 [1, 57]	10.6 ± 6.5 [1,28]	6.5 ± 3.5 [1,12]
Time spent on travel from home to the clinic and from the clinic to home during in-person visits over five years (hours)	52.2 ± 42.2 [0.8, 284.1]	49.4 ± 35.8 [0.8, 181.4]	63.5 ± 54.1 [1.1, 284.1]	26.6 ± 25.4 [2.5, 87.7]
Total time spent on in-person visits over five years (hours)^a^	79.5 ± 50.2 [5.0, 343.7]	76.9 ± 42.8 [5.0, 238.6]	91.2 ± 62.5 [5.0, 343.7]	45.7 ± 28.1 [9.9, 108.2]
Total working time lost over five years (hours)	65.9 ± 39.7 [4.6, 251.5]	65.4 ± 35.4 [4.6, 210.3]	71.4 ± 44.8 [5.0, 220.7]	36.3 ± 17.8 [8.7, 71.5]
Total travel cost over five years (Euros)	461.4 ± 374.6 [3.0, 2143.1]	440.9 ± 329.7 [3.0, 2042.4]	556.3 ± 456.8 [6.0, 2143.1]	207.0 ± 211.3 [10.1, 696.9]
**Data for ten years**				
Number of in-person visits over ten years	21.0 ± 14.0 [2, 115]	21.2 ± 13.3 [1, 115]	21.2 ± 13.0 [2, 55]	13.0 ± 7.0 [2, 24]
Time spent on travel from home to the clinic and from the clinic to home during in-person visits over ten years (hours)	104.4 ± 84.3 [1.5, 568.2]	98.8 ± 71.6 [1.5, 362.8]	127.0 ± 108.3 [2.3, 568.2]	53.1 ± 50.7 [5.1, 175.3]
Total time spent on in-person visits over ten years (hours)^a^	157.8 ± 100.1 [10.1, 685.8]	152.6 ± 85.4 [11.5, 475.6]	181.1 ± 124.6 [11.5, 685.8]	90.4 ± 55.7 [19.7, 214.9]
Total working time lost over ten years (hours)	131.3 ± 79.4 [9.2, 501.5]	130.3 ± 70.8 [9.2, 420.6]	142.5 ± 89.6 [11.5, 441.3]	72.2 ± 35.6 [17.4, 142.9]
Total travel cost over ten years (Euros)	922.7 ± 749.1 [6.0, 4286.2]	881.7 ± 659.5 [6.0, 4084.9]	1112.6 ± 913.6 [11.9, 4286.2]	414.0 ± 422.7 [20.3, 1393.7]

Data are n (%) or mean ± standard deviation [minimum, maximum].

^a^This includes time of travelling from home to the clinic, time spent at the clinic and time of travelling home from the clinic.

### Secondary endpoints

The average travel cost of patients with DBS and their caregivers per visit was 50.4 ± 28.8 Euros.

Analyzing data of 298 patients, total travel costs for the first year, five years, and ten years after the activation were found 151.8 ± 108.7 Euros, 461.4 ± 374.6 Euros, and 922.7 ± 749.1 Euros, respectively. However, in some cases, these costs can even be 726.3 Euros, 2143.1 Euros, and 4286.2 Euros for the first year, five years, and ten years, respectively. Subgroup data for secondary endpoints can be found in Table 2.

### Tertiary endpoints

Comparing data of PD and dystonia patients, we found that subjects with dystonia had to travel longer distances to our unit (244.7 ± 141.0 km vs. 197.7 ± 122.5 km; *p* = 0.039), however, travel time from home to the clinic did not differ significantly (197.3 ± 106.8 min vs. 163.7 ± 92.4 min; *p* = 0.073). Travel cost per in-person visit was also higher for dystonia patients (58.2 ± 30.6 Euros vs. 48.4 ± 27.2 Euros; *p* = 0.039). Despite higher travel costs, patients with dystonia had more in-person visits in the first year (3.7 ± 1.9 vs. 3.2 ± 1.7; *p* = 0.039), and consistently, first-year data showed higher travel costs for them (190.0 ± 130.0 Euros vs. 141.8 ± 97.2 Euros; *p* = 0.004). The number of in-person visits did not differ significantly for ten years (21.2 ± 13.0 vs. 21.2 ± 13.3; p = 0.713), but travel costs remained higher for dystonia patients (1112.6 ± 913.6 Euros vs. 881.7 ± 659.5 Euros; *p* = 0.044).

Comparing data of PD and ET patients, no significant differences were detected in travel distances (197.7 ± 122.5 km vs. 175.8 ± 164.1 km; *p* = 0.459) and times (163.7 ± 92.4 min vs. 153.9 ± 133.8 min; *p* = 0.644). Consequently, travel cost per in-person visit was also comparable (48.4 ± 27.2 Euros vs. 41.5 ± 37.1 Euros; *p* = 0.304). However, patients with PD had more in-person visits in the first year (3.2 ± 1.7 vs. 2.4 ± 1.5; *p* = 0.032), and consistently, first-year travel costs were found higher for them (141.8 ± 97.2 Euros vs. 88.5 ± 85.2 Euros; *p* = 0.030). Both the number of in-person visits and travel costs for ten years remained higher for PD patients (21.2 ± 13.3 vs. 13.0 ± 7.0, *p* = 0.012 and 881.7 ± 659.5 Euros vs. 414.0 ± 422.7 Euros, *p* = 0.005, respectively).

Compared to ET patients, subjects with dystonia had to travel longer distances to our unit (244.7 ± 141.0 km vs. 175.8 ± 164.1 km; *p* = 0.037), however, travel time from home to the clinic did not differ significantly between the groups (197.3 ± 106.8 min vs. 153.9 ± 133.8 min; *p* = 0.151). Travel cost per in-person visit was found higher for dystonia patients (58.2 ± 30.6 Euros vs. 41.5 ± 37.1 Euros; *p* = 0.033). Despite higher travel costs, patients with dystonia had more in-person visits in the first year (3.7 ± 1.9 vs. 2.4 ± 1.5; *p* = 0.010), and consistently, first-year travel costs were also higher for them (190.0 ± 130.0 Euros vs. 88.5 ± 85.2 Euros; *p* = 0.004). Both the number of in-person visits and travel costs for ten years remained higher for dystonia patients (21.2 ± 13.0 vs. 13.0 ± 7.0, *p* = 0.014 and 1112.6 ± 913.6 Euros vs. 414.0 ± 422.7 Euros; *p* < 0.001).

We found a negative correlation between travel distance from home to our unit and the number of first-year in-person visits (Spearman’s rho = −0.269, *p* < 0.001) that became somewhat stronger regarding the number of in-person visits over ten years (Spearman’s rho = −0.314, *p* < 0.001). Patients living ≤ 50 km from our unit (n = 56) had more frequent in-person visits during the first year and over ten years after the activation than those (n = 306) living further than 50 km (4.3 ± 2.3 vs. 2.7 ± 1.7, p < 0.001 and 28.9 ± 18.1 vs. 17.4 ± 12.3, *p* < 0.001).

Number of visits during the first year did not differ significantly between patients aged ≤ 65 years (n = 283) and over (n = 79) 65 years (3.0 ± 1.9 vs. 2.7 ± 1.8; *p* = 0.271). However, although subjects aged over 65 years did not live further from our unit compared to patients ≤ 65 years (188 ± 121 km vs. 219 ± 129 km; *p* = 0.061), they had less in-person visits over ten years after the activation (19.7 ± 13.4 vs. 17.1 ± 15.8; *p* = 0.036). Numbers of in-person visits in the first year and over ten years after the activation did not differ significantly for patients with an education level of ≤ 12 years (n = 215) and over (n = 147) 12 years (2.9 ± 1.8 vs. 3.0 ± 2.1, *p* = 0.868 and 18.6 ± 14.6 vs. 20.2 ± 12.8, *p* = 0.090).

Regarding PD, there were negative correlations between both the HYS and the motor score of the MDS-UPDRS recorded at the DBS activation visit and the numbers of in-person visits over ten years (Spearman’s rho = −0.293, *p* < 0.001 and Spearman’s rho = −0.267, *p* < 0.001, respectively). Patients with HYS > 2 PD (n = 89) had less frequent in-person visits than those with HYS ≤ 2 PD (n = 150) both during the first year and over ten years (2.6 ± 1.8 vs. 3.0 ± 1.9, *p* = 0.041 and 14.4 ± 11.1 vs. 22.4 ± 13.8, *p* < 0.001, respectively). Using previously determined cut-off value for the Motor Part of the MDS-UPDRS between mild and moderate PD (32/33 points)^15^, number of first year in-person visits did not differ significantly (2.9 ± 1.9 vs. 2.8 ± 1.9; *p* = 0.460), however, patients with moderate or severe PD (n = 131) had less frequent in-person visits over ten years than subjects with mild PD (n = 108) at the activation visit (16.7 ± 11.5 vs. 22.7 ± 14.9, *p* < 0.001).

Regarding dystonia, we found a negative correlation between the BFMDDS scores recorded at DBS activation and the numbers of in-person visits over ten years (Spearman’s rho = −0.284, *p* = 0.009). Number of first year in-person visits did not differ significantly between patients with ≥ 5 points on the BFMDDS (n = 56) and those with less disability (n = 27) at DBS activation (3.1 ± 1.9 vs. 3.8 ± 2.1; *p* = 0.219), however, patients with a disability of ≥ 5 points on the BFMDDS had less frequent in-person visits over ten years (17.8 ± 12.2 vs. 23.8 ± 13.8, *p* = 0.037).

Regarding ET, there was no correlation between the summary index of the QUEST and numbers of in-person visits in the first year and over ten years after DBS activation (Spearman’s rho = 0.049, *p* = 0.826 and Spearman’s rho = 0.240, *p* = 0.270, respectively). Considering the cut-off values for the summary index of the QUEST determining different levels of ET severity^16^, we found no significant differences among patients representing different levels of disease severity.

To determine the effects of COVID-19 pandemic on DBS care, data of 234 patients could be used. Comparing the total number of all (in-person and telemedicine) visits 18 months just before and after the outbreak of COVID-19 pandemic, we found a significant decrease (3.7 ± 2.1 vs. 2.4 ± 2.7; *p* < 0.001). To this, the decrease in the frequency of in-person visits (3.6 ± 2.0 vs. 1.7 ± 1.8; *p* < 0.001) could be the main contributor. In contrast, the use of telemedicine care and its proportion of all visits increased significantly (0.1 ± 1.3 vs. 0.5 ± 1.2, *p* < 0.001 and 3.9 ± 7.5% vs. 17.6 ± 27.7%, *p* < 0.001, respectively, Table 3).

**Table 3:** Number of in-person and telemedicine visits before and after the outbreak of COVID-19 pandemic.

	Number of all (in-person and telemedicine) visits in the half and a year before the outbreak of COVID-19 pandemic	Number of all (in-person and telemedicine) visits in the half and a year after the outbreak of COVID-19 pandemic	*p*-value
All (n = 234)	3.7 ± 2.1 [0, 20]	2.4 ± 2.7 [0, 20]	< 0.001
Parkinson’s disease (n = 148)	3.9 ± 2.0 [0, 20]	2.6 ± 2.7 [0, 20]	< 0.001
Dystonia (n = 63)	3.5 ± 1.9 [1,8]	1.8 ± 2.3 [0, 13]	< 0.001
Essential tremor (n = 10)	2.6 ± 0.9 [1,4]	1.1 ± 1.5 [0, 5]	0.003

	Number of in-person visits in the half and a year before the outbreak of COVID-19 pandemic	Number of in-person visits in the half and a year after the outbreak of COVID-19 pandemic	
All (n = 234)	3.6 ± 2.0 [0, 17]	1.7 ± 1.8 [0, 13]	< 0.001
Parkinson’s disease (n = 148)	3.7 ± 1.9 [0, 17]	1.7 ± 1.6 [0, 10]	< 0.001
Dystonia (n = 63)	3.3 ± 1.9 [1,8]	1.3 ± 1.6 [0, 7]	< 0.001
Essential tremor (n = 10)	2.5 ± 0.8 [1,4]	1.1 ± 1.1 [0, 4]	0.002

	Number of telemedicine visits in the half and a year before the outbreak of COVID-19 pandemic	Number of telemedicine visits in the half and a year after the outbreak of COVID-19 pandemic	
All (n = 234)	0.1 ± 1.3 [0, 3]	0.5 ± 1.2 [0, 10]	< 0.001
Parkinson’s disease (n = 148)	0.2 ± 0.4 [0, 3]	0.9 ± 1.8 [0, 10]	< 0.001
Dystonia (n = 63)	0.2 ± 0.3 [0, 1]	0.5 ± 1.0 [0, 6]	0.001
Essential tremor (n = 10)	0.1 ± 0.1 [0, 1]	0.1 ± 0.3 [0, 1]	1.000

	Percentage of telemedicine visits in the half and a year before the outbreak of COVID-19 pandemic	Percentage of telemedicine visits in the half and a year after the outbreak of COVID-19 pandemic	
All (n = 234)	3.9 ± 7.5% [0.0%, 45.5%]	17.6 ± 27.7% [0.0%, 100.0%]	< 0.001
Parkinson’s disease (n = 148)	4.1 ± 7.8% [0.0%, 45.5%]	19.9 ± 28.7% [0.0%, 100.0%]	< 0.001
Dystonia (n = 63)	3.9 ± 6.8% [0.0%, 27.8%]	16.4 ± 28.2% [0.0%, 100.0%]	< 0.001
Essential tremor (n = 10)	1.3 ± 3.2% [0.0%, 10.5%]	1.8 ± 6.0% [0.0%, 20.0%]	1.000

Data are mean ± standard deviation [minimum, maximum].

## Discussion

Although there is an increasing body of research into telecare including remote DBS programming, an emerging novel technology^4–10^, our knowledge is still lacking on several aspects of teleprogramming. In this study, we aimed to explore potential clinical and economic advantages of DBS teleprogramming in Hungary focusing on MDs.

The majority of patients treated with DBS at our unit are nonlocal residents. We found that our patients generally have to travel 208 km to get to our unit which usually takes almost 3 h, while the mean total travel cost per in-person visit is 50.4 Euros, however, in some cases, these can also reach 544 km, 9 h, and 119.5 Euros, respectively. These numbers are smaller than those found by a recent Chinese study^8^, however, regarding this, the difference in the territory of the two countries (93,030 vs. 9,596,961 square kilometers) should also be considered. Assuming that all adjustments after the activation can be made remotely, according to our results on travel costs, DBS teleprogramming could generally save at least 151.8 and 922.7 Euros for patients in the first year and over a ten-year treatment period, respectively, which are about the half and three times the Hungarian net minimum monthly wage, however, these numbers could also be 726.3 and 4286.2 Euros (about a two-monthly and fourteen-monthly Hungarian net minimum wage, respectively). In addition, 3 and 17 days holiday could be generally saved in the first and over ten years which can be important for actively working patients and caregivers who accompany the patients for on-site visits.

In addition to suspected economic benefits of remote programming, our results also shed light on potential clinical advantages of DBS telecare. At our department, the in-person examination time is generally 20 min per patient. During a detailed status assessment which is performed at least annually in our protocol, in addition to a physical neurological examination during the basic 20-min follow-up, further disease-specific scales measuring the motor and non-motor symptoms, the disorder-related disability and the health-related quality of life (e.g., the MDS-UPDRS, the BFMDRS, the Fahn-Tolosa-Marin Tremor Rating Scale, the Beck Depression Inventory, the Montreal Cognitive Assessment, the 39-item Parkinson’s Disease Questionnaire, etc.) are also assessed. The basic physical neurological examination and additional tests generally last for 120 min. As a result, for several patients, a greater part of time spent on an in-person visit is not related to the examinations rather to other activities such as travelling which may demotivate patients in visiting to DBS centers. Therefore, teleprogramming could also improve patient compliance at least with annual detailed status assessments—considering that almost all parts of the physical neurological examination and most of the scales used to measure severity of MDs can be performed via video-based consultation which is included in a teleprogramming session—but also with more frequent check-ups. Increased patient compliance might also have beneficial effects on the efficacy of DBS treatment.

According to a recent analysis, televisits have been mainly performed via phone calls and mostly for drug prescriptions in Hungary^17^. However, studies have found that video-based consultation has several advantages over telephone such as fewer medication errors, greater diagnostic accuracy, and improved decision-making accuracy^18^. This tool is feasible for assessing all main motor symptoms of MDs with the exemption of muscle tone and postural stability^19,20^. In addition, it has been found that patients also prefer video-based consultation over phone calls^21,22^. One reason for the wider use of phone calls compared to video-based consultation can be that the latter requires a more extensive technical background, therefore, its installations and maintenance are more expensive. Because video-based consultation is a fundamental part of the NeuroSphere™ DBS teleprogramming system, in countries where present neurological telemedicine practice is similar to the Hungarian one (e.g., in Norway)^23^, remote programming could also help to increase general efficacy of telecare.

The present study also tried to determine the groups of patients who could particularly benefit from teleprogramming. Travel distance negatively correlated with the number of in-person visits both on short and long term, however, just after the activation and as the disease progresses, more frequent DBS adjustments may be necessary to maintain desired symptomatic control and quality of life^24,25^. We found that patients living further than 50 km from the DBS center could benefit from teleprogramming by having more regular DBS adjustments in the early post-activation period and during the disease course. These findings are in line with the results of another recent study^26^, however, it should be also noted that telemedicine usage does not necessarily correlate with travel distance according to other trials^27^, which might imply that independently of travel distance, all patients can derive benefit from improved access to healthcare due to telemedicine. This aspect should be further clarified in future trials.

Additionally, patients with dystonia had to travel the longest distance for in-person visits, however, despite longer travels, they had the highest numbers of in-person visits both in the first year and over ten years after DBS activation. Consequently, travel costs were the highest for dystonia patients both on short and long term. These results may be partly explained by the fact that only few centers provide dystonia care of which our unit is the main DBS center for dystonia in Hungary with an extensive referral network, therefore, a large portion of patients had to travel long distances to have DBS therapy regularly optimized. In addition, after adjustments of DBS of dystonia patients, immediate symptomatic response cannot often be observed and as a result, more frequent programming may be necessary. Considering that our finding supports the results of a previous survey that found the accessibility of dystonia experts difficult in several European countries^28^, teleprogramming could also help improving accessibility of DBS care for dystonia.

In our study, patients aged over 65 years or having ≥ 33 points on the Motor Part of the MDS-UPDRS and ≥ 5 points on the BFDDS at DBS activation travelled less frequently to our unit over ten years. The main explanation for this may be that travelling can be challenging for these patients due to mobility problems resulting from higher age, more severe symptoms, and disease-related disability. However, closer follow-up and regular DBS adjustment would be essential mainly for this patient group. These subjects may on the one hand be more dependent on their caregivers, if they are available, in travelling to their medical appointments which can substantially increase caregiver burden. On the other hand, in case caregivers are lacking, patients may become unable to travel for in-person visits. Considering this, teleprogramming could be useful not just from economic perspectives, but could also help achieving better symptomatic control and higher quality of life and decreasing caregiver burden.

This analysis revealed that during COVID-19, on-site patient care significantly decreased compared to the period prior to the pandemic, however, there was an impressive increase in the requests for telemedicine care. Although telemedicine has had several advantages, DBS adjustments could not been performed remotely in Hungary. Thus, with the reduction in the number of in-person visits, there has been a risk for a decrease in the effectiveness of DBS treatment and as a result, a deterioration in the condition of patients^29,30^. Considering this, teleprogramming could also help maintaining a more secure delivery of DBS therapy. According to an ongoing survey at our center, a high portion of DBS patients surveyed thus far (63 subjects out of 75, 84.0%) would try remote programming independently of (n = 45, 71.4%) or due to (n = 18, 28.6%) the COVID-19 pandemic. This is in line with the results of a recent survey by the Parkinson’s Foundation and Abbott Labs^26^.

This study might have some limitations. First, because we analyzed data only on the Hungarian healthcare system, the outcomes should be used with caution in other countries. Second, the study might have also been limited by its single-center design and consequent effects of center-specific characteristics on the outcomes. For example, possible differences in the protocols of DBS care including remote strategies among centers may be present. In our practice, allowing amplitude modification and change between at least two different programming groups are standards of care, while in some other centers, patients may not be allowed any programming options. However, because our unit is a tertiary MD center, our patient pool covers the whole area of the country, therefore, we could collect and analyze data on the potential clinical and economic benefits of remote DBS programming on a nation-wide level. Another strength of this analysis can be the high number of included patients representing different types and severity of MDs. In addition, analyses were performed not just for the whole study population but also the main disorder subgroups, and we could analyze data from all waves of the COVID-19 pandemic period. At this point, it should also be mentioned that our unit is the main DBS center for dystonia, while DBS for ET is more equally available in Hungarian centers. As a result, patients with dystonia may be overrepresented while ET subjects underrepresented in our analyses. Possibly lower portion of patients with ET and higher portion of dystonia subjects relative to other units from other countries together with the fact that ET usually shows a good response to DBS treatment even after initial programming settings and less visits are therefore required for adjustments, should also be considered during the interpretation of our findings. options. During the interpretation of our findings, it should also be considered that Last but not least, because the cost per patient is a modeled estimate and not based on any actual data reported by patients, some costs that may occur for some patients (e.g., cost of accommodation for patients cannot travel to site and back to home on the same day due to the long travel distance) or possibility of public transport were not taken into account during the analyses which may also limit the universal applicability of our approach.

To conclude, by revealing several potential clinical and economic advantages of remote DBS programming, our study supports the introduction of teleprogramming in Hungary. With careful interpretation, our findings may be useful for other countries that also plan to introduce DBS remote programming. Patients who live further from the DBS center, are of older age, have dystonia or represent higher level of disease severity and disability could particularly benefit from this technical innovation. However, further analyses from other countries for which our study can be a good basis and data from clinical practice using remote programming could provide a more comprehensive picture of the role and value of DBS telemedicine.

## Data Availability

The data used to support the findings of this study have not been made available because the current ethical approval does not permit their deposition. The datasets are available from the corresponding author on reasonable request after the approval of the relevant ethical committees.

## Abbreviations

BFMDDS: Burke–Fahn–Marsden Dystonia Disability Scale
DBS: Deep brain stimulation
ET: Essential tremor
HYS: Hoehn and Yahr Scale
MD: Movement disorder
MDS-UPDRS: Movement Disorder Society-sponsored Unified Parkinson’s Disease Rating Scale
PD: Parkinson’s disease
